# Psychometric Properties of the Thai Version Psychological Well-Being Scale and the Factors Related to among Thai Patients with Major Depressive Disorder

**DOI:** 10.1155/2021/2592548

**Published:** 2021-01-29

**Authors:** Nanchatsan Sakunpong, Kwanjai Ritkumrop

**Affiliations:** Behavioral Science Research Institute, Bangkok 10110, Thailand

## Abstract

**Background:**

The assessment to provide care and support to patients with major depressive disorder (MDD) currently focuses on the recovery from the disease, but it is still lacking in measuring and developing psychological well-being among Thai patients with MDD. Therefore, this research is aimed at studying the psychometric properties of the Thai version psychological well-being scale and study factors related to among patients with MDD.

**Materials and Methods:**

The Thai version psychological well-being scale, an 8-point Likert-type scale, was translated by our research team and used to examine psychometric properties as well as to identify the factors related to psychological well-being in a cross-sectional study among samples of 111 patients diagnosed with MDD from Princess Maha Chakri Sirindhorn Medical Center in Nakhon Nayok Province, Thailand.

**Results:**

Cronbach's alpha for the Thai version psychological well-being scale was .91, unidimensionality was examined with exploratory factor analysis, and the structural validity was assessed with confirmatory factor analysis. The convergent validity was found using the correlation coefficients of the Thai version psychological well-being scale with the Beck Depression Inventory (BDI) and Gratitude Questionnaire (GQ-6). However, none of the social factors were significantly correlated with Thai patients' psychological well-being with MDD.

**Conclusion:**

The Thai version psychological well-being scale is a brief and concise scale with high reliability to evaluate Thai patients with MDD which can support and improve their well-being.

## 1. Introduction

The prevalence of major depressive disorder (MDD) has been found at a high rate globally, and in Thailand [1]. The problems caused by the disease ranged from living impairments daily and due to loss of life from suicide [2]. Psychologists, which are one of the most important professions to treat patients with MDD, have been consistently conducting extensive research to expand knowledge of and insight into this area. However, most studies focused only on the causes, related factors, and procedures for treating patients with MDD. In addition, the study related to well-being among patients with MDD is still lacking, and the findings have been scarce. The study focused on well-being does not only view MDD patients in a positive light, and it was developed to increase their growth and happiness, associated with the definition of mental health provided by the World Health Organization (WHO)

“Mental health is a state of well-being in which an individual realizes his or her own abilities, can cope with the normal stresses of life, can work productively and is able to make a contribution to his or her community” [3].

In psychology, the interest in happiness or well-being has only emerged over the past decades. The currently available studies on well-being can be divided into two major groups: subjective and psychological well-being. The subjective well-being consists of two subgroups: (1) emotional process—the presence of positive emotions and the absence of negative emotions; (2) cognitive process, which represents through life satisfaction [4]. Psychological well-being can be described as a positive characteristic related to growth, the meaning of life, and life purpose [5]. According to Ryff [6] and Ryff and Singer [7], psychological well-being consists of self-acceptance, purpose in life, positive relationships with others, environmental mastery, personal growth, and autonomy. The main difference between subjective well-being and psychological well-being is that subjective well-being involves short-term emotions [8], while psychological well-being focuses on in-depth personal experience [9, 10] as well as multidimensional functions covering other aspects of well-being. While there is a growing interest to develop the psychological well-being scale, the available ones consisted of numerous items; for example, the psychological well-being scales by Deci and Ryan [11] and Ryff [6]. Therefore, there is a need to develop a brief and comprehensive scale. The brief scale of the psychological well-being scale by Diener et al. or flourishing scale [12–14] consisted of 8 items, covering social and psychological aspects of well-being such as positive relationships, self-esteem, and living with meaning and purpose. It is a Likert scale ranged from 1 to 7, strong disagreement to strong agreement. The scale was evaluated in a sample of university students in the United States to measure psychometric properties and found Cronbach's alpha reliability at .86 and convergent validity with Ryff's psychological well-being scale from the correlation coefficient at .80. The construct validity was examined with exploratory factor analysis, revealing one strong factor with an eigenvalue above 1.0. Thus, this brief scale can be used to measure social-psychological well-being properly. From previous studies, the psychological well-being scale has been translated into 17 languages used to measure social-psychological well-being among several samples such as student samples in the United States [15], employee samples in Portugal [16], community samples in China [17] general population in the Netherlands [13, 14], and older adult samples in Iran [18]. However, no study has assessed the psychometric properties of this scale among Thai patients with MDD before.

In this study, the research team translated the scale into Thai and evaluated the scale's psychometric properties among Thai patients with MDD which can be considered a cross-cultural study. As a result, the Thai clinician can use this scale to explore, measure, and provide feedback and plan the intervention to develop social-psychological well-being among Thai patients with MDD. In addition, this study also is aimed at examining the social factors related to social-psychological well-being among MDD patients supporting Thai psychologists and clinicians to identify risk groups with poor well-being who may need attentive care, not only to recover from depression but also to develop their personal growth.

## 2. Materials and Methods

This research is a cross-sectional study. According to this study's objectives, the research team divided the study into two phases: (1) to evaluate psychometric properties of and (2) to study the factors related to social-psychological well-being among MDD patients. The samples in this study included 111 MDD patients diagnosed by a psychiatrist and met the DSM-IV-TR criteria at Princess Maha Chakri Sirindhorn Medical Center in Nakhon Nayok Province, Thailand. The samples were chosen with purposive sampling and treated at the psychiatric outpatient department from the 1st of March 2019 until the 25th of February 2020 and participated voluntarily in this research. They all had the literacy skills required to read, write, and complete all scale items. Due to confirmatory factor analysis to examine the psychometric properties of this study, the sample size was calculated based on the criteria that considered the proportions of the observable variables (item number) per sample at 1 : 10-20 [19]. For the full 8-item scale, the appropriate sample size of 80 (8 × 10) people was considered. However, to prevent insufficient data, the researcher collected data from a total of 111 samples and found that all 111 forms were completed.

### 2.1. Instruments

The instruments in this research consisted of 4 different measures, as follows:

1. The Thai version of psychological well-being scale—the research team translated the brief psychological well-being scale by Diener et al. [12], with permission to publicly publish, and references to the source. The scale consists of 8 items to assess social-psychological well-being in critical dimensions, such as positive relationships, feelings of competence, and having meaning and purpose in life. It ranges from 1 to 7 on a scale from strong disagreement to strong agreement. This scale has a Cronbach's alpha coefficient value of .86, with a construct validity based on the study among samples in the United States
2. The Beck Depression Inventory—to measure depression in this research, the Beck Depression Inventory is used. Pulpipat [20] translated and developed the self-report inventory, which consisted of 21 question items in a 4-rating scale, which measured symptoms of depression, related to their behavior, feelings, and emotions in the past week. The research team has reevaluated the inventory's reliability among the 111 MDD patients in this study and found high internal consistency, with Cronbach's alpha coefficients of .93
3. The Gratitude Questionnaire by McCullough et al. [21] has been translated by Chinkijkarn et al. [22]. A short self-report of 6 items on a 5-rating scale was used in this study. The researchers examined the reliability of the questionnaire among the samples of 111 MDD patients with the Cronbach's alpha coefficient and found an internal consistency of .77
4. Personal information questionnaire was utilized to determine the social factors related to the social-psychological well-being of MDD patients. This personal information questionnaire is aimed at inquiring about personal details including gender, age, marital status, monthly income, debt, educational level, duration of psychiatric treatment, physical illness, and psychiatric medication usage duration

### 2.2. Procedure

1. The translation of the scale from English to Thai (forward translation) was completed by two native Thai translators in the research team. One translator graduated with a doctoral degree, and the other is currently completing their doctorate in psychology. They have acquired knowledge and expertise in conceptual theory related to the variables of well-being. Both translators independently translated the scale prior to a discussion leading to a consensus on the translation
2. Backward translation from Thai to English was completed by 2 different translators from the forward translation group in procedure (1). This group of translators consisted of native Thai people, who had completed both degrees in English or Teaching English as a Foreign Language and Psychology in bachelor's degree, master's degree, or doctoral degree. Furthermore, they had never seen or read the original English manuscript of the scale
3. The translation team in procedures (1) and (2) mutually considered the semantic and conceptual consistency between the backward translation, from Thai to English, and the original English manuscript. Any differences in translation or editing were subject to debate and finalized through a consensus agreement
4. The Thai revised version reached a consensus agreement among translation teams was trialled with patients at the SWU Clinic who were diagnosed with MDD following the criteria of DSM-IV-TR. The patients completed the scale, followed by cognitive interviews to verify their understanding of the items and word selection in the scale. The patients' suggestions were used to improve the scale accordingly and to finalize the completed Thai version psychological well-being scale
5. The questionnaire, inventory, and scales were used to collect information from 111 samples to examine the psychometric properties of the Thai version of the psychological well-being scale and to determine the factors related to social-psychological well-being among MDD patients

### 2.3. Statistical Analysis

1. The item analysis was assessed using the corrected item-total correlation
2. The convergent validity between the Thai version of the psychological well-being scale, the Gratitude Questionnaire, and the Beck Depression Inventory was examined using Pearson's correlation analysis
3. The unidimensionality of the Thai version of the psychological well-being scale was statistically analyzed by exploratory factor analysis, factor extraction by principal component analysis (PCA), and under the consideration of the components with an eigenvalue greater than 1
4. The construct validity was assessed by confirmatory factor analysis
5. The examination of multiple mean difference was completed by the Analysis of Variance (ANOVA) statistics, and the different comparison was analyzed with Scheffe statistics

## 3. Results

The research results were divided into three sections: Section 3.1 is the general information of the sample, Section 3.2 is the research findings in response to the research objective 1, and Section 3.3 is the research findings regarding research objective 2.

### 3.1. General Information

The sample of a total of 111 patients diagnosed with MDD by psychiatrists was mostly female (81%), aged between 18 and 35 years old (52%), single (54%), an average monthly income between 10,000 and 30,000 baht (41%), in debt (47%), graduated with a Bachelor's degree or higher (54%), had no physical illnesses (51%), and taking antidepressant medication for less than one year (55%).

### 3.2. Psychometric Properties of the Thai Version Psychological Well-Being Scale

The psychometric properties of the scale can be explained in detail, as follows:

1. The item analysis with corrected item-total correlation score ranges was between .651 and .756
2. The unidimensionality examined with exploratory factor analysis and extracted by principal component analysis displayed only a single factor with an eigenvalue greater than 1.0, accounting for up to 61% of the total variance. This represents the internal consistency of all items under a single component
3. The reliability of the full scale by using Cronbach's alpha coefficient was .91
4. The validity of the Thai version psychological well-being scale was measured by the Pearson correlation coefficient, with the Beck Depression Inventory and the Gratitude Questionnaire which were at -.34 and .30, respectively, and with a statistical significance at .05. This indicates that the Thai version of the psychological well-being scale has convergent validity. The construct validity was examined with confirmatory factor analysis, and it was found that the psychological well-being scale model was consistent with the empirical data (*χ*^2^/d*ƒ* = 1.3, GFI = .95, AGFI = .90, CFI = .99, SRMR = .03, RMSEA = .05). All eight items have factor loading between .69 and .80 and with a statistical significance of .05

The psychometric properties of the Thai version psychological well-being scale are summarized in Table 1.

### 3.3. Factors Related to Social-Psychological Well-Being in Patients with MDD

When comparing the mean scores of social-psychological well-being based on social factors of the samples in terms of gender, age, marital status, average monthly income, amount of debt, highest education level, underlying physical disease, and antidepressant usage duration, no factor was found to have a statistically significant correlation with the level of social-psychological well-being among MDD patients, as detailed in Table 2.

## 4. Discussion

From this study, it was found that the Thai version psychological well-being scale had a high-reliability score of .91 [23–27] even with only eight items, and acceptable corrected item-total correlation. This result can be explained that each of the eight items was internally consistent, reflecting the theoretical structure of social-psychological well-being [26, 27]. The evaluation of the psychometric properties by collecting data from all 111 samples with a diverse background, in terms of gender, age, marital status, monthly income, debt, highest educational level, underlying physical disease, and duration of antidepressant medication usage, supported the reason why this scale had high reliability [26, 27]. Also, the unidimensionality of the scale examined by exploratory factor analysis showed that the scale has only one factor which can be referred to as the high homogeneity of the scale [19]. Confirmatory factor analysis was used to evaluate the validity of the scale, and all of the items had factor loading between .69 and .80, with a statistical significance of .05. The psychological well-being model among patients with MDD was also consistent with the empirical data (*χ*^2^/d*ƒ* = 1.3, GFI = .95, AGFI = .90, CFI = .99, SRMR = .03, RMSEA = .05) which reflected the construct validity of each item on the scale. The Thai version of the psychological well-being scale also has convergent validity, as measured by the Pearson correlation with the Beck Depression Inventory and the Gratitude Questionnaire. The result from convergent validity may be explained that because people with low levels of well-being have a high risk of developing depression in the future [28] and well-being is a variable inversely correlated to depression along the same continuum [29]. In addition, gratitude can be explained as a positive emotion, an important component of well-being [30, 31]. From these reasons, depression and gratitude are associated with well-being theoretically.

In terms of the factors related to social-psychological well-being, it has been found that gender, age, marital status, average monthly income, debt, highest educational level, underlying physical disease, and the duration of antidepressant medication usage were not significantly correlated. This may be because the samples in this study were patients with MDD. There may be biological factors related; i.e., neurotransmitter dysfunctions, genetics, and psychological factors; i.e., deep cognitive structure [2]. On the other hand, the factors in this study were mainly social factors that may not explain the levels of psychological well-being among these samples. However, further studies are needed to determine which factors can relate to the development of social-psychological well-being among MDD patients.

## 5. Limitation of the Study

This study was not without limitation. For example, the samples in this study were selected from only one medical center in the central region of Thailand, which may not be well representative of all patients with MDD. Therefore, this study's result, especially the factors related to social-psychological well-being, could be confirmed by assessing participants from other hospitals or areas of Thailand to more accurately reflect patients with major depressive disorder within the Thailand culture.

## 6. Conclusion

When providing care and treatment to patients with MDD, the focus should not rely solely on the recovery from the disease. The patient's social-psychological well-being development, which indicates more comprehensive care, should also be implemented. The Thai version psychological well-being scale is an alternative for exploring, screening, providing feedback, and planning intervention to develop social-psychological well-being for Thai MDD patients. This scale is a short, concise form with a high level of reliability and validity examined by Thai MDD samples. Therefore, the Thai version of a psychological well-being scale is a reliable measure used in clinical practice to develop more comprehensive mental health care for Thai MDD patients.

## Figures and Tables

**Table 1:** Overview of the psychometric properties of the Thai version psychological well-being scale classified by each item.

ข้อคำถาม	CITC	*λ*	SE	*t*	*R* ^2^
(1) ฉันมีจุดมุ่งหมายในชีวิตและใช้ชีวิตอย่างมีความหมาย (I lead a purposeful and meaningful life)	.73	.78	-^∗∗^	-^∗∗^	.62
(2) สัมพันธภาพทางสังคมที่ฉันมีนั้น ช่วยเกื้อหนุนและส่งเสริม อีกทั้งเป็นเหมือนรางวัลให้กับชีวิต (my social relationships are supportive and rewarding)	.65	.71	.12	7.73^∗^	.50
(3) ฉันให้ความสำคัญและสนใจในกิจกรรม ต่างๆ ของฉันที่ทำในแต่ละวัน (I am engaged and interested in my daily activities)	.70	.76	.12	8.40^∗^	.58
(4) ฉันให้ความใส่ใจที่จะสร้างความสุขและความเป็นอยู่ที่ดีขึ้นให้กับผู้อื่น (I actively contribute to the happiness and well-being of others)	.70	.72	.12	7.87^∗^	.52
(5) ฉันมีศักยภาพและความสามารถในการทำกิจกรรมต่างๆที่มีความสำคัญกับฉัน (I am competent and capable in the activities that are important to me)	.76	.80	.11	8.99^∗^	.64
(6) ฉันเป็นคนดีและมีชีวิตที่ดี (I am a good person and live a good life)	.74	.72	.12	7.86^∗^	.52
(7) ฉันมองอนาคตของฉันในเชิงบวก (I am optimistic about my future)	.74	.75	.12	8.30^∗^	.56
(8) ผู้คนให้ความเคารพในตัวฉัน (people respect me)	.65	.69	.12	7.35^∗^	.47
Overall reliability by Cronbach's alpha = .91					

^∗^Means *p* value less than .05. ^∗∗^Means constrain factor loading equal to 1.00.

**Table 2:** Factors related to social-psychological well-being among MDD patients (*n* = 111).

Factor	*n* (percent)	Mean	SD	*p* value
Gender				.38
Female	90 (81.1)	40.40	9.33	
Male	13 (11.7)	42.31	11.76	
Gender diversities	8 (7.2)	36.38	7.95	
Age (years)				.14
12-18	8 (7.2)	40.38	8.19	
19-35	58 (52.3)	38.91	9.82	
36-60	35 (31.5)	40.94	9.54	
More than 60	10 (9.0)	46.40	7.18	
Marital status				.21
Single	60 (54.1)	38.88	9.55	
Marriage	33 (29.7)	42.42	9.39	
Divorce/separate/widow	18 (16.2)	41.33	9.54	
Monthly income (Thai baht)				49
Less than 10,000	43 (38.7)	39.40	10.17	
10,000–30,000	45 (40.5)	40.20	9.53	
More than 30,000	23 (20.8)	42.35	8.39	
Debt				.57
Yes	52 (46.8)	40.88	10.60	
Denied	59 (53.2)	39.85	8.57	
Highest educational level				.23
Uneducated or primary school	22 (20.0)	43.41	10.35	
Secondary school or equivalent	26 (23.4)	39.15	10.18	
Bachelor degree or above	63 (56.7)	39.75	8.90	
Underlying physical disease				.33
Yes	54 (48.6)	41.24	9.33	
Denied	57 (51.4)	39.47	9.75	
Duration of antidepressant usage (year)				.52
Less than 1	61 (55.0)	39.46	8.59	
1-3	48 (43.2)	41.52	10.67	
More than 3	2 (1.8)	38.50	10.61	

## Data Availability

All the data included in the manuscript can be accessed from the corresponding author Nanchatsan Sakunpong upon request through the email address of nanchatsans@gmail.com
